# A clinical, morphological and molecular study of 70 patients with gastrointestinal involvement in systemic mastocytosis

**DOI:** 10.1038/s41598-023-49749-z

**Published:** 2024-01-06

**Authors:** Johannes Lübke, Nicole Naumann, Oliver Hoffmann, Hans-Peter Horny, Karl Sotlar, Martina Rudelius, Georgia Metzgeroth, Alice Fabarius, Wolf-Karsten Hofmann, Andreas Reiter, Juliana Schwaab

**Affiliations:** 1 Department of Hematology and Oncology, University Hospital Mannheim, Heidelberg University, Theodor-Kutzer-Ufer 1-3, 68167 Mannheim, Germany; 2 Department of Pathology, Ludwig-Maximilians-University, Munich, Germany; 3 Department of Pathology, Paracelsus Medical University of Salzburg, Salzburg, Austria

**Keywords:** Myeloproliferative disease, Gastrointestinal diseases

## Abstract

In 70 patients with *KIT* D816V positive systemic mastocytosis (SM) including 36 patients with advanced SM (AdvSM), we correlated the extent of reported mucosal mast cell ([m]MC) infiltration of the upper and/or lower gastrointestinal tract (UGIT, n = 63; LGIT, n = 64; both, n = 57) with symptoms and markers of MC burden/subtype. GI symptoms were reported by all patients (mean 2.1 number of symptoms). A strong mMC infiltration was identified in 24 patients (UGIT, 17/63, 27%; LGIT, 19/64, 30%). Concurrent involvement of UGIT and LGIT (n = 12) correlated with female gender (75%) and a higher symptom burden (mean 2.7) but not with MC burden or subtype. Significant differences between non-AdvSM and AdvSM were reported regarding food intolerance (54% vs. 17%), cramping (54% vs. 22%) and weight loss (0% vs. 64%). *KIT* D816V was identified in 54/56 (96%) available biopsies. In 46 patients, digital PCR revealed a correlation with low albumin levels (r =  − 0.270, *P* = 0.069) and the *KIT* D816V VAF in peripheral blood (r = 0.317, *P* = 0.036) but not with the extent of mMC infiltration or markers of MC burden/subtype. Although MC mediator triggered GI symptoms have a substantial impact on the quality of life, correlation to objective disease parameters is lacking thus making its systematic assessment challenging.

## Introduction

Systemic mastocytosis (SM) is a rare myeloid neoplasm characterized by variable infiltration and multifocal accumulation of neoplastic mast cells (MC) in bone marrow (BM), skin and visceral organ systems^1–5^. According to the International Consensus Classification (ICC)/World Health Organization Classification (WHO-5), the major diagnostic criterion for SM is the presence of MC aggregates (defined as 15 or more MCs) in BM or other extracutaneous organs, including the gastrointestinal tract (GIT)^4,5^. Minor diagnostic criteria include co-expression of CD25/CD2/CD30 by neoplastic MCs, 25% of MCs with a spindle-shaped or atypical morphology, the presence of an activating point mutation at codon 816 of KIT (in ≥ 90% *KIT* D816V, driver mutation), and a serum total tryptase > 20 ng/mL (ICC: in absence of a myeloid neoplasm; WHO-5: adjusted in case of hereditary alpha-tryptasemia). Advanced SM (AdvSM) comprises the subtypes aggressive SM (ASM), SM with an associated myeloid neoplasm (SM-AMN), and MC leukemia (MCL)^4^. Indolent phases of the disease include indolent SM (ISM), bone marrow mastocytosis (BMM; low BM MC infiltration and tryptase and absence of cutaneous involvement) and smoldering SM (BM MC infiltration > 30% and serum tryptase > 200 µg/L)^1,5,6^.

MC mediator release, e.g. through histamines, leukotrienes, and organ infiltration lead to manifold symptoms including life-threatening complications^7,8^. GI symptoms are present in up to 50–70% of SM patients and include, but are not limited to food intolerance, nausea, emesis, cramping, and diarrhea, ultimately causing malabsorption/weight loss representing a C-finding for diagnosis of ASM^1,9–12^. Although GI involvement constitutes a fundamental factor for morbidity and quality of life, only little is known about its association with the overall clinical, morphological and genetic features.

We therefore sought to investigate the presence and extent of reported gastrointestinal mucosal MC infiltration and analyze the correlation with symptoms, markers of disease burden and subtype in 70 SM patients.

## Methods

### Patients

Initially, a total of 246 *KIT* D816V positive SM patients with any signs of GI symptoms (presence or absence of food intolerance, nausea/emesis, cramping, diarrhea, weight loss > 10%) were identified in the ‘German Registry on Disorders of Eosinophils and Mast Cells’ (GREM). For further analysis, the mean number of symptoms per patient was assessed and correlated to laboratory and histopathological findings. Diagnosis and subtyping of SM were carried out according to the ICC criteria^4^. The study design adhered to the tenets of the Declaration of Helsinki and was approved by the institutional review board of the Medical Faculty of Mannheim, Heidelberg University (Heidelberg, Germany). All patients gave written informed consent.

### Evaluation of biopsies

Only patients with a histopathological documentation of their GI biopsies were enrolled in this study. The median number of biopsies conducted per patient was 11 (range 3–26). For descriptive analyses, we semi-quantified the extent of MC infiltration in GI biopsies by categorizing them into “strong” MC infiltration (if histologic reports described either compact or diffuse dense infiltrates) versus “minor” MC infiltration (diffuse scattered infiltrates described in the reports). A diagnostic work-up of biopsies was considered to be complete, if a full immunohistochemical staining including KIT, CD25 and MC tryptase was performed and the MC count per high-power field or percentage of MC infiltration in relation to the evaluated area were indicated. Evaluations were carried out at local site or by reference pathologists from the European Competence Network on Mastocytosis (ECNM; H.-P. Horny and K. Sotlar). All BM biopsies and BM smears were evaluated by the ECNM reference pathologists.

### Chip-based digital PCR

Measurements of the *KIT* D816V VAF on DNA, derived from peripheral blood (PB) and biopsies, were performed using the QuantStudio™ three-dimensional (3D) dPCR system in combination with the Applied Biosystems ProFlex PCR System (ThermoFisher Scientific, Waltham, MA, USA). Per sample, a 15 µL reaction volume was prepared. The volume included 7.1 µL of 10 ng/µL DNA, 7.5 µL of QuantStudio™ 3D Digital PCR Master Mix v2 (ThermoFisher Scientific, Waltham, MA, USA) and 0.4 µL of *KIT* D816V specific Taqman gene expression assay (ID: Hs000000039_rm, ThermoFisher Scientific Waltham, MA, USA).

### Statistics

Clinical, laboratory, morphological and molecular data were collected at time of diagnosis. The Mann–Whitney *U*-test was used to compare continuous variables and medians of distributions. For categorical variables, Fisher’s exact test was carried out. The Spearman rank correlation was applied as a nonparametric measure of rank correlation. *P* values of < 0.05 (two-sided) were considered statistically significant. Data management and statistical analyses were performed with SPSS (SPSS version 20.0; IBM Corporation, Armonk, NY, USA) and GraphPad Prism software (version 8, GraphPad, La Jolla, CA, USA).

## Results

### Patients and GI infiltration pattern

**Figure 1:**
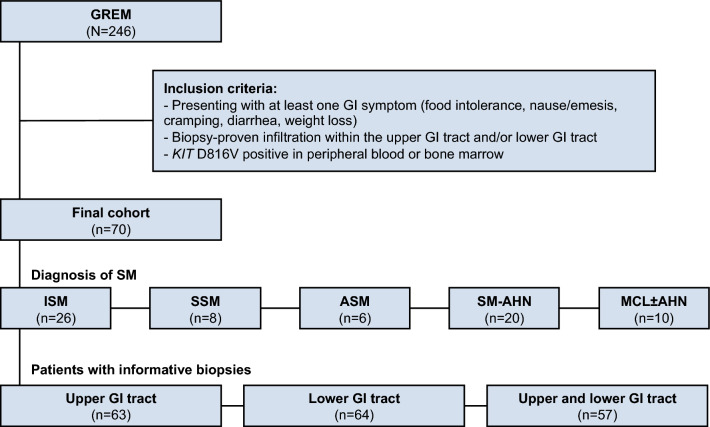
Patient flowchart. *AdvSM* advanced systemic mastocytosis, *ASM* aggressive systemic mastocytosis, *GI* gastrointestinal, *GREM* German Registry on Disorders of Eosinophils and Mast Cells, *ISM* indolent systemic mastocytosis, *MCL* ± *AHN* mast cell leukemia with/without an associated hematologic neoplasm, *SM* systemic mastocytosis, *SM-AHN* systemic mastocytosis with an associated hematologic neoplasm, *SSM* smoldering systemic mastocytosis.

We identified 70 patients with *KIT* D816V positive SM (ISM, n = 26, 37%; SSM, n = 8, 11%; ASM, n = 6, 9%; SM-AHN, n = 20, 29%; MCL ± AHN, n = 10, 14%), GI symptoms and reported mucosal MC infiltration of the upper (UGIT, n = 63) and/or lower GIT (LGIT, n = 64; Fig. 1). UGIT infiltration was observed in 19/30 (63%) ISM/SSM and 25/33 (76%) AdvSM patients. Stomach (ISM/SSM, 11/30, 37%; AdvSM 17/33, 52%) and duodenum (ISM/SSM, 16/30, 53%; AdvSM 24/33, 73%) were frequently involved, while no esophageal infiltration was not reported in any subtype. LGIT infiltration was prominent in both ISM/SSM (31/33, 97%) and AdvSM (29/31, 97%) patients and mostly affected the terminal ileum (ISM/SSM, 29/33, 88%; AdvSM 29/31, 94%) while colon involvement was observed in 16/33 (48%) ISM/SSM and 20/31 (65%) AdvSM patients (Table 1).

**Table 1:** Gastrointestinal involvement among the different segments of the upper and lower gastrointestinal tract.

UGIT (n = 63)	ISM/SSM n = 30		AdvSM n = 33		*P*
	*n*	%	*n*	%	
Esophagus	0	0	0	0	–
Stomach	11	37	17	52	0.312
Duodenum	16	53	24	73	0.125
Stomach and duodenum	8	27	16	48	0.119
Overall	19	63	25	76	0.410

LGIT (n = 64)	ISM/SSM n = 33		AdvSM n = 31		*P*
	*n*	%	*n*	%	
Terminal ileum	16	48	20	65	0.218
Colon	29	88	29	94	0.673
Ileum und Colon	14	42	20	65	0.087
Overall	31	97	29	97	0.999

*AdvSM* advanced systemic mastocytosis, *ISM* indolent systemic mastocytosis, *n* number, *SSM* smoldering systemic mastocytosis.

### GI symptoms

Symptoms were reported by all patients with a mean number of 2.1 symptoms (range 1–5) per patient: food intolerance (25/70, 36%), nausea/emesis (20/70, 29%), cramping (25/70, 36%), diarrhea (57/70, 81%) and weight loss (23/70, 33%). There was no statistically significant association between GI symptoms and any particular location of GI involvement (*P* > 0.500). The comparisons of symptoms in relation to minor vs. strong mucosal infiltration, ISM vs. AdvSM and various levels of *KIT* D816V VAF are summarized in Tables 2, 3 and 4.

**Table 2:** Frequency of gastrointestinal symptoms within indolent and advanced systemic mastocytosis.

	ISM n = 26		SSM n = 8		AdvSM n = 36		*P*
	*n*	*%*	*n*	*%*	*n*	*%*	
Weight loss ≥ 10%	0	0	0	0	23	64	< 0.001
Nausea/emesis	8	31	3	38	9	25	0.496
Abdominal cramping	14	54	3	38	8	22	0.015
Diarrhea	22	85	7	88	28	78	0.419
Food intolerance	14	54	5	63	6	17	0.001
Presence ≥ 2 symptoms	15	58	4	50	25	69	0.241
Presence ≥ 3 symptoms	12	46	3	38	11	31	0.241
Presence ≥ 4 symptoms	6	23	3	38	4	11	0.099

*AdvSM* advanced systemic mastocytosis, *ISM* indolent systemic mastocytosis, *n* number, *SSM* smoldering systemic mastocytosis, *y/n* yes/no.

The *P* values refer to the Mann–Whitney *U* test comparing non-advanced systemic mastocytosis (ISM and SSM) versus AdvSM.

**Table 3:** Frequency of gastrointestinal symptoms within patients with biopsies of minor and strong mucosal mast cell infiltration.

	Minor mucosal MC infiltration^a^ n = 46		Strong mucosal MC infiltration^a^ n = 24		*P*
	*n*	*%*	*n*	*%*	
Weight loss ≥ 10%	13	28	10	42	0.589
Nausea/emesis	11	24	9	38	0.232
Abdominal cramping	18	39	7	29	0.409
Diarrhea	36	78	21	88	0.345
Food intolerance	16	35	9	38	0.822
Presence ≥ 2 symptoms	26	57	18	75	0.128
Presence ≥ 3 symptoms	16	35	10	42	0.572
Presence ≥ 4 symptoms	8	17	5	21	0.725

*MC* mast cell, *n* number, *y/n* yes/no.

^a^Strong mucosal mast cell infiltration: compact or diffuse dense infiltrates.

The *P* values refer to the Mann–Whitney *U* test.

**Table 4:** Frequency of gastrointestinal symptoms depending on the *KIT* D816V variant allele frequency of gastrointestinal biopsies.

	VAF < 5% n = 18		VAF 5–10% n = 9		VAF ≥ 10% n = 19		*P*
	*n*	*%*	*n*	*%*	*n*	*%*	
Weight loss ≥ 10%	5	28	2	22	5	26	0.943
Nausea/emesis	8	44	4	44	8	42	0.886
Abdominal cramping	12	67	5	56	8	42	0.859
Diarrhea	14	78	7	78	16	84	0.138
Food intolerance	10	56	5	56	10	53	0.825
Presence ≥ 2 symptoms	12	67	7	78	15	79	0.793
Presence ≥ 3 symptoms	10	56	6	67	10	53	0.805
Presence ≥ 4 symptoms	8	44	2	22	3	16	0.619

*n* number, *y/n* yes/no, *VAF* variant allele frequency.

The *P* values refer to the Mann–Whitney *U* test comparing VAF < 5%/VAF 5–10% versus VAF ≥ 10%.

#### Histological and immunohistochemical evaluation

A complete immunohistochemical staining (tryptase, CD117, CD25) was performed in 76/127 biopsies (UGIT, 35/63, 56%; LGIT, 41/64, 64%). An exact quantitative assessment of MC density (MC count per high-power field or percentage of MC infiltration) was available for 18/127 (14%) biopsies only (Table 5).

**Table 5:** Evaluation of gastrointestinal involvement in 70 patients with systemic mastocytosis.

	UGIT	LGIT
Number of patients, *n* (%)	63 (70)	64 (71)
Diagnostic work-up of pathologist		
Tryptase stain available, *n* (%)	39 (62)	46 (72)
Positivity, *n* (%)	35 (90)	44 (96)
CD117 stain available, *n* (%)	47 (75)	54 (84)
Positivity, *n* (%)	44 (94)	51 (94)
CD25 stain available, *n* (%)	42 (67)	51 (80)
Positivity, *n* (%)	26 (62)	42 (82)
Mast cell infiltration level available, *n* (%)	9 (14)	9 (14)
Infiltration level, %; median (range)	25 (20–30)	20 (10–30)
PCR available, *n* (%)	25 (40)	50 (78)
Positivity, *n* (%)	22 (88)	49 (98)
Strong mucosal mast cell infiltration^a^, *n* (%)	17 (27)	19 (30)
GI site involvement vice versa, *n* (%)	12 (71)	12 (63)
Further work-up of biopsies		
Chip-based digital PCR available, *n* (%)	8 (13)	38 (59)
Variant allele frequency, %; median (range)	4.6 (0.2–47.2)	2.7 (0.0–49.9)

*GI* gastrointestinal, *LGIT* lower gastrointestinal tract, *n* number, *UGIT* upper gastrointestinal tract, *PCR* polymerase chain reaction.

^a^Strong mucosal mast cell infiltration: compact or diffuse dense infiltrates.

Compact infiltrates were defined according to the ICC classification^4^, either being micronodular or band-like in a subepithelial position. Band-like infiltrates were present in the mucosal layer of the GI tract in the patients with positive biopsies.

Diffuse MC infiltrates usually showed the following features:

1. A significant increase in MCs throughout the lamina propria mucosae, > 25% of them with a spindle-shaped appearance.
2. A reduced expression of tryptase but (usually weak) CD25 (or CD 30).

Because of the frequent occurrence of numerous intermingled eosinophils, scattered diffuse infiltrates were difficult to identify without immunohistochemistry.

### Severity of GI involvement

A strong MC infiltration (compact or diffuse dense infiltrates) was identified in 24 patients (UGIT, 17/63, 27%; LGIT, 19/64, 30%). It was concurrently observed in UGIT and LGIT in 12/57 (21%) patients of which 5 and 7 patients had non-AdvSM (ISM, n = 4; SSM, n = 1) or AdvSM, respectively (Table 6). If present at one site, the probability to also detect it at the other site was 71% and 63%, respectively (Table 5). The concurrent strong MC infiltration was associated with female gender (9/12, 75%) and a higher number of symptoms (mean 2.7).

**Table 6:** Demographical and disease characteristics of 12 patients with strong mucosal mast cell infiltration within the upper and lower gastrointestinal tract.

#	Sex	Age in years at Dx of SM	WHO Dx	Type of SM Type of AHN	A/T	H/S	Serum tryptase (µg/L)	MCI in BM (%)	Albumin (mg/dL)	Ferritin (µg/L)	Alkaline phosphatase (U/I)	*KIT* D816V VAF in PB (%)	*KIT* D816V VAF in biopsies (%)	Weight loss^b^	Frequency of symptoms^c^
8	M	71	SM-AHN	MCL ± MDS/MPNu	+/−	− / +	870	30	23		126	3		+	++
11	F	65	ASM	–	−/−	+/−	186	20	32	178	115	22	42	+	++
21	M	73	SM-AHN	MCL ± MDS/MPNu	−/−	+ / +	377	35	39		575	18	13	+	+++
25	M	46	ASM	–	−/−	+/−	194	35	41		154	18		+	++
30	F	44	SM-AHN	ISM ± MDS/MPNu	−/−	−/−	150	10	35	100	98	3	19	−	+++
40	F	69	SM-AHN	MCL ± CMML	+/−	+ / +	354	40	39	442	246	5		+	++
48	F	48	ASM	–	+/−	−/−	62	20	22		36	20		+	++
37	F	63	ISM	–	−/−	−/−	18								+
2	F	43	SSM	–	−/−	−/−	302	75	40		119	8	30	−	++++
51	F	73	ISM	–	−/−	−/−	36	15	44	140	82	0.4	1	−	++++
9	F	50	ISM^a^	–	−/−	−/−	25	10	43		98	11^a^	4	−	++++
69	F	31	ISM^a^	–	−/−	−/−	54	10	46	27	72	26^a^	6	−	+++

Data obtained at time of diagnosis.

*AHN* associated hematologic neoplasm, *ASM* aggressive systemic mastocytosis, *A/T* anemia < 10.0 g/dL (+), > 10.0 g/dL (−), platelets < 100 × 10^9^/L (+), > 100 × 10^9^/L (−), *BM* bone marrow, *CMML* chronic myelomonocytic leukemia, *Dx* diagnosis, *F* female, *H/S* palpable hepatomegaly with impairment of liver function, ascites and/or portal hypertension (+), if not (−), palpable splenomegaly with hypersplenism (+), if not (−), *ISM* indolent systemic mastocytosis, *M* male, *MCI* mast cell infiltration, *MDS/MPN-U* myelodysplastic/myeloproliferative neoplasm, unclassified, *PB* peripheral blood, *SM* systemic mastocytosis, *SSM* smoldering systemic mastocytosis, *VAF* variant allele frequency, *WHO* World Health Organization.

^a^With multilineage involvement without histomorphological diagnosis of an AHN. Patients did also not meet criteria for SSM according to the revised WHO 2022 classification.

^b^Weight loss ≥ 10% in 6 months (+), if not (−).

^b^The following symptoms were considered for analysis: food intolerance, nausea/emesis, cramping, diarrhea, weight loss > 10%.

### Qualitative and quantitative (chip-based) PCR for *KIT* D816V

**Figure 2:**
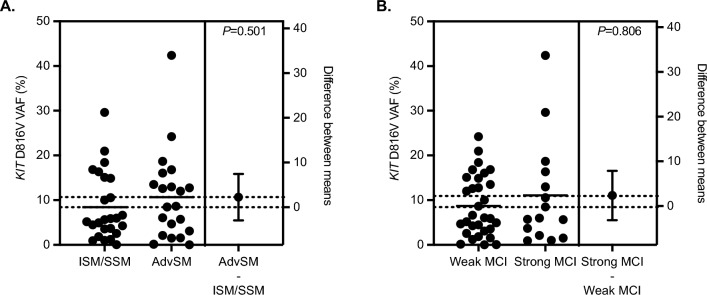
*KIT* D816V variant allele frequency performed of gastrointestinal biopsies to (**A**) diagnosis and (**B**) extent of mast cell infiltration. *AdvSM* advanced systemic mastocytosis, *ISM* indolent systemic mastocytosis, *MCI* mucosal mast cell infiltration, *SSM* smoldering systemic mastocytosis.

Qualitative PCR on DNA extracted and purified from UGIT and/or LGIT GI biopsies was performed for 56/70 (80%) patients of which 54/56 (96%) were *KIT* D816V positive (Table 5). Chip-based quantitative dPCR was carried out for 46 patients. Neither the median *KIT* D816V VAF nor the grouping of the *KIT* D816V VAF (< 5% [n = 18, 39%]), 5–10% [n = 9, 20%], > 10% [n = 19, 41%]) indicated an association with symptoms (Table 4), mucosal MC infiltration (strong vs. minor, *P* = 0.806; Fig. 2B), parameters of MC burden (Table 7) or subtype (non-AdvSM vs. AdvSM, *P* = 0.501; Fig. 2A). Spearman correlations revealed a positive correlation of the *KIT* D816V VAF from GI biopsies with the *KIT* D816V VAF in PB (ρ = 0.317, *P* = 0.036) and a negative correlation with the albumin level (ρ = − 0.270, *P* = 0.069) (Table 8).

**Table 7:** Demographic and disease characteristics of patients with systemic mastocytosis according to *KIT* D816V VAF of gastrointestinal biopsies.

*KIT* D816V VAF in GI biopsies	< 5%	5–10%	> 10%	*P*
Number of patients at baseline, *n* (%)	18 (39)	9 (20)	19 (41)	–
Age at diagnosis; median (range)	52 (33–73)	53 (31–73)	45 (34–73)	0.660
Male, *n* (%)	7 (39)	3 (33)	4 (21)	0.483
Advanced systemic mastocytosis, *n* (%)	7 (39)	4 (44)	10 (53)	0.686
C-findings				
Hemoglobin, g/dL; median (range)	13 (7–16)	14 (9–15)	13 (8–15)	0.798
< 10 g/dL, *n* (%)	2 (11)	2 (22)	2 (11)	0.408
Platelets, × 10^9^/L; median (range)	255 (12–361)	178 (88–397)	241 (65–497)	0.384
< 100 × 10^9^/L, *n* (%)	4 (22)	2 (22)	1 (5)	0.998
AP, U/L; median (range)	89 (40–621)	74 (55–365)	99 (47–1206)	0.830
> 150 U/L, *n* (%)	4 (22)	2 (22)	5 (26)	0.815
Albumin level, g/L; median (range)	41 (26–47)	41 (33–46)	39 (30–44)	0.080
< 34 g/L, *n* (%)	3 (17)	1 (11)	5 (26)	0.360
B-findings				
MC-infiltration in BM, %; median (range)	25 (5–60)	20 (10–30)	18 (5–100)	0.628
Serum tryptase, µg/L; median (range)	100 (14–925)	70 (25–1030)	150 (10–1382)	0.660
Splenomegaly, *n* (%)	10 (56)	5 (56)	10 (53)	1.000
Hepatomegaly, *n* (%)	9 (50)	4 (44)	9 (47)	0.785
Lymphadenopathy, *n* (%)	9 (50)	6 (67)	12 (67)	0.500
Other relevant findings				
Leukocytes, × 10^9^/L; median (range)	8.1 (3.2–29.4)	7.0 (2.7–13.1)	8.7 (3.6–79.3)	0.455
LDH, U/L; median (range)	156 (105–224)	137 (69–170)	168 (117–653)	0.616
*KIT* D816V VAF in PB, %, median (range)	1 (0–49)	4 (0–46)	21 (0–49)	0.095

*AP* alkaline phosphatase, *BM* bone marrow, *GI* gastrointestinal, *LDH* lactate dehydrogenase, *MC* mast cell, *n* number, *PB* peripheral blood, *VAF* variant allele frequency.

The *P* values refer to the Jonckheere–Terpstra test or the Cochran-Armitage test comparing patients with < 5%, ≥ 5–10% and ≥ 10% *KIT* D816 VAF in gastrointestinal biopsies.

**Table 8:** Spearman correlation of *KIT* D816V variant allele frequency performed of gastrointestinal biopsies with several laboratory and histological parameters.

	Spearman correlation ρ	*P*
*KIT* D816V VAF in PB	0.317	0.036
BM MC-infiltration	− 0.072	0.640
Leukocytes	0.104	0.490
Hemoglobin	0.013	0.934
Platelets	0.142	0.347
Albumin	− 0.270	0.069
Cholesterol	− 0.011	0.944
Triglyceride	− 0.144	0.345
Creatinine	0.036	0.812
Alkaline phosphatase	− 0.019	0.901
Lactate dehydrogenase	0.111	0.464
Tryptase	0.110	0.465

*BM* bone marrow, *MC* mast cell, *PB* peripheral blood, *VAF* variant allele frequency.

## Discussion

Beside hepatic MC infiltration with impaired liver function and splenomegaly, mucosal MC infiltration of the GI tract mediates a heterogeneous clinical scenario of mild to severe GI symptoms including food intolerance, nausea, vomiting, abdominal cramping, diarrhea and anaphylaxis. Most severe cases are characterized by malabsorption and significant weight loss > 10% which are established C-findings. Several studies have reported a highly variable GI symptom burden in up to 50–70% of SM patients^9–12^. However, the real frequency may be underestimated, because many patients do not undergo an adequate diagnostic work-up on basis of a multi-disciplinary approach. Causes for this may include non-performance of endoscopy, non-detection of microscopic MC infiltration in macroscopically non-suspicious mucosa or the misdiagnosis of eosinophilic colitis due to the inadequate use of immunohistochemistry^9,10,13–16^.

In our series, diarrhea was the most commonly reported subtype-independent symptom, while weight loss was most frequently observed in AdvSM and food intolerance and abdominal cramping in ISM/SSM. A concurrent strong mucosal MC infiltration in UGIT and LGIT was identified in 21% of patients and in either UGIT or LGIT in further 13% of patients. A strong mucosal MC infiltration in one or both regions was independent of symptoms, markers of MC burden or subtype. In fact, 42% of those patients had ISM/SSM highlighting a discordant quantitative MC infiltration of BM and GI. These findings imply considerations at which level of mucosal MC infiltration and associated severe symptoms, e.g. life-threatening anaphylaxis, purely symptomatic treatment with antihistamines, MC stabilizers and local/systemic corticosteroids should be complemented by targeted treatment with KIT inhibitors in carefully selected patients even in the absence of a formal diagnosis of AdvSM^17–20^.

We also sought to assess to which extent the qualitative or quantitative measurement of the *KIT* D816V VAF in GI biopsies through dPCR may provide additional useful information. VAF levels were not correlated with the extent of MC infiltration or markers of disease burden. There was only a moderate correlation with low albumin levels and the *KIT* D816V VAF in PB with the *KIT* D816V VAF in GI biopsies, the latter even suggesting a potential mix of GI tissue and blood thus highlighting obvious flaws of the techniques applied. Therefore, as opposed to its undisputed diagnostic, prognostic and even predictive value in PB^21–25^, a routine implementation of a quantitative *KIT* D816V VAF testing in GI tract biopsies seems not to have an additional benefit in the diagnostic work up of SM-related GI involvement. However, the histological staining with tryptase, CD117 and CD25 as well as the presence of compact MC infiltrates are of utmost relevance in discriminating between reactive and neoplastic conditions, as MCs are usually also found in normal GI tract mucosa and can be markedly elevated in patients with inflammatory bowel disease. Even a positive staining for CD25 does not always allow a conclusive diagnosis of minor GI SM involvement as it also stains a subpopulation of background lymphocytes in other conditions. It may therefore be of limited value in the discrimination between low GI infiltration and reactive MC increase^26^. Earlier studies on the clinical correlation of GI tract infiltration and clinical symptoms also could not identify a correlation of clinical symptoms with the pattern and degree of MC infiltration indicating that the MC mediator release rather than the direct infiltration of the GI tract by MCs is responsible for the clinical symptoms^9,10^. Furthermore, the assessment and quantification of MC infiltration in GI samples is to some extent arbitrary and subject to a high degree of interindividual variation. MC thresholds for the different sections of the GI tract like the ones for eosinophils in hypereosinophilic conditions do not exist given the limitations of MC assessment as outlined above. In patients with unexplained GI symptoms, exclusion of an underlying SM should therefore not only be performed by endoscopy but should also include a serum tryptase screening and a BM analysis at least in patients with elevated levels. If SM diagnosis is excluded, patients with unexplained high levels of serum tryptase and GI symptoms should be evaluated for hereditary alpha-tryptasemia (HαT)^27^. Diagnosis of HαT and SM was concurrently seen in 2/2 patients tested within our population. Both patients had high objective disease parameters as well as a high disease burden, showing that HαT positivity is not restricted to SM negative cases but otherwise no further conclusions can be drawn given the small number of analyzed samples.

### Limitations

This study focused solely on the mere presence or absence of the five symptoms outlined in the methodology, omitting consideration of additional symptoms potentially associated with SM such as constipation, bloating, flatulence, feeling of fullness, loss of appetite, dysphagia, or gastrointestinal bleeding. Consequently, the selected symptoms in this study may not comprehensively capture the spectrum of GI manifestations in SM, potentially leading to the oversight of patients experiencing more severe gastrointestinal symptoms. Individual symptoms were not quantitatively assessed, precluding a more comprehensive analysis upon the association of symptom and GI disease burden. In terms of the techniques employed, we observed a moderate correlation between the *KIT* D816V VAF in PB and GI biopsies, suggesting the possibility of a mixture of GI tissue and blood and methodically flaws. Additionally, the evaluation and quantification of MC infiltration in GI samples exhibit a degree of subjectivity and substantial interindividual variation as outlined above. Taken together, these findings underline the difficulties in assessment of GI tract involvement of SM. A multidisciplinary approach in SM patients with suspected GI involvement seems prudent. Whereas the assessment of GI involvement can be important to establish a diagnosis of SM, due to the rate of false positive results and possible misinterpretations by less experienced pathologists, undirected diagnostics with multiple stainings in patients without otherwise proven SM should be avoided. Histopathological assessment of the BM should therefore remain the diagnostic mainstay for detection of the disease in patients with suspected SM diagnosis.

## Conclusions

Although GI manifestations in SM patients are highly prevalent and often disabling, clinical symptoms do not correlate with histologic findings of disease course or disease subtyping. MC mediator related diarrhea and malabsorption remain challenging to quantify but might be directly related to suboptimal absorption and bioavailability (pre-systemic elimination) of drug therapies in patients with SM and should therefore be taken into account.

## Data Availability

The datasets used and/or analyzed during the current study are available from the corresponding author on reasonable request.
